# Visceral adiposity is associated with metabolic profiles predictive of type 2 diabetes and myocardial infarction

**DOI:** 10.1038/s43856-022-00140-5

**Published:** 2022-07-01

**Authors:** Javeria Raheem, Eeva Sliz, Jean Shin, Michael V. Holmes, G. Bruce Pike, Louis Richer, Daniel Gaudet, Tomas Paus, Zdenka Pausova

**Affiliations:** 1 The Hospital for Sick Children, University of Toronto, Toronto, ON Canada; 2 Departments of Physiology and Nutritional Sciences, University of Toronto, Toronto, ON Canada; 3 MRC Population Health Research Unit at the University of Oxford, Oxford, OX3 7LF UK; 4 Department of Radiology and Clinical Neurosciences, University of Calgary, Calgary, AB Canada; 5 Department of Health Sciences, Université du Québec à Chicoutimi, Chicoutimi, QC Canada; 6 Clinical Lipidology and Rare Lipid Disorders Unit, Community Genetic Medicine Center, Department of Medicine, Université de Montréal, Montreal, QC Canada; 7 ECOGENE-21, Chicoutimi, QC Canada; 8 Departments of Psychiatry and Neuroscience, Centre Hospitalier Universitaire Sainte-Justine, Universite de Montreal, Montreal, QC Canada; 9 Departments of Psychology and Psychiatry, University of Toronto, Toronto, ON Canada

**Keywords:** Obesity, Dyslipidaemias

## Abstract

**Background:**

Visceral fat (VF) increases risk for cardiometabolic disease (CMD), the leading cause of morbidity and mortality. Variations in the circulating metabolome predict the risk for CMD but whether or not this is related to VF is unknown. Further, CMD is now also present in adolescents, and the relationships between VF, circulating metabolome, and CMD may vary between adolescents and adults.

**Methods:**

With an aim to add understanding to the metabolic variations in visceral obesity, we tested associations between VF, measured directly with magnetic resonance imaging, and 228 fasting serum metabolomic measures, quantified with nuclear magnetic resonance spectroscopy, in 507 adults (36–65 years) and 938 adolescents (12–18 years). We further utilized data from published studies to estimate similarities between VF and CMD-associated metabolic profiles.

**Results:**

Here we show that VF, independently of body mass index (BMI) or subcutaneous fat, is associated with triglyceride-rich lipoproteins, fatty acids, and inflammation in both adults and adolescents, whereas the associations with amino acids, glucose, and intermediary metabolites are significant in adults only. BMI-adjusted metabolomic profile of VF resembles those predicting type 2 diabetes in adults (*R*^2^ = 0.88) and adolescents (*R*^2^ = 0.70), and myocardial infarction in adults (*R*^2^ = 0.59) and adolescents (*R*^2^ = 0.40); this is not the case for ischemic stroke (adults: *R*^2^ = 0.05, adolescents: *R*^2^ = 0.08).

**Conclusions:**

Visceral adiposity is associated with metabolomic profiles predictive of type 2 diabetes and myocardial infarction even in normal-weight individuals and already in adolescence. Targeting factors contributing to the emergence and maintenance of these profiles might ameliorate their cumulative effects on cardiometabolic health.

## Plain language summary

Obesity increases risk for multiple diseases, likely due to changes in how the body breaks down carbohydrates and fats and due to the molecules that circulate in the blood as a result. In obesity, the accumulation of fat around the internal organs of the abdomen (visceral fat) may be more detrimental than fat accumulation elsewhere in the body. Here, we show that visceral fat is associated with variations in the levels of various molecules in the blood, and these variations are similar to those that can predict type 2 diabetes and heart disease, not only in adults but also in adolescents. This suggests that visceral fat accumulation may be closely related to development of these diseases from a young age.

## Abstract (web summary)

Raheem, Sliz et al. study the serum metabolome and an imaging-based measure of visceral fat in adults and adolescents. The authors find that, independently of BMI, visceral fat quantity is associated with a metabolomic profile similar to those predictive of type 2 diabetes and myocardial infarction.

## Introduction

Obesity has emerged as a global health problem in adults as well as youth^1,2^. Excess adiposity increases the risk for multiple disorders, including cardiometabolic disease (CMD)^3^. Obesity and CMD are innately related with multiple aberrations in the levels of circulating metabolites: metabolomic studies have shown that obesity is causally related to deviations in blood lipids, glucose, and amino acids^4^, and that similar metabolic deviations predict the development of CMD including type 2 diabetes (T2D) and incident cardiovascular disease^5–7^.

The distribution of body fat is a key modulator of obesity-related risk for CMD; individuals who store body fat within the abdominal cavity rather than elsewhere in the body are at greater risk^8,9^. The previous metabolomic studies have estimated obesity indirectly using body mass index (BMI)^4^ and there is only a limited number of reports directly assessing visceral fat (VF)^10^. Further, the previous studies predominantly investigated adult populations^10^ and the evidence in youth is lacking.

To add understanding to VF-related changes in systemic metabolism, we investigated the associations between magnetic resonance imaging (MRI)-quantified VF volume and blood metabolome in a two-generational study of adolescents (average age 15 years) and adults (average age 49 years). We further tested VF-by-age group interactions for each metabolite to examine the impact of age group on the associations. In secondary analyses, we tested for sex-specific models as previous literature suggests that there are sex differences in VF accumulation^11^, in the circulating metabolome^12^, as well as CMD prevalence and mortality^13^. Finally, to provide insights into whether similar metabolic pathways may be involved in VF and CMD, we compared the metabolomic association profile of VF with those predicting T2D, myocardial infarction (MI), and ischemic stroke (IS) reported in published studies^6,7^.

Our results suggest that VF, independently of general obesity estimated by BMI, associates with aberrations in lipid metabolism in both generations and, in adults only, also with aberrations in amino acid and glucose metabolism. These VF-associated metabolomic variations highly resemble those predicting T2D and MI, which further suggest that visceral adiposity may be detrimental for cardiometabolic health not only in adults but also in adolescents.

## Methods

### Study population

Here we studied 956 adolescents (12–19 years of age) and 598 parents (36–65 years of age) from the Saguenay Youth Study, a two-generational population-based cohort from the Saguenay-Lac Saint-Jean region of Quebec, Canada (details described in ref. ^14^). Individuals with self-reported lipid disease (15 adolescents and 14 adults) or on lipid-lowering medications (3 adolescents and 77 adults) were excluded from the analyses. The final sample included 938 adolescents and 507 adults. All adults and adolescents provided written consent and assent, respectively. The study was approved by the Research Ethics Boards of the Integrated Health and Social Services Centres of the Saguenay-Lac St. Jean, Chicoutimi, Canada (2002-023, 2003-005, and 2011-008) and of the Hospital for Sick Children in Toronto, Canada (#1000032154 and #1000068660).

### Measurements

#### Adiposity

**Fig. 1:**
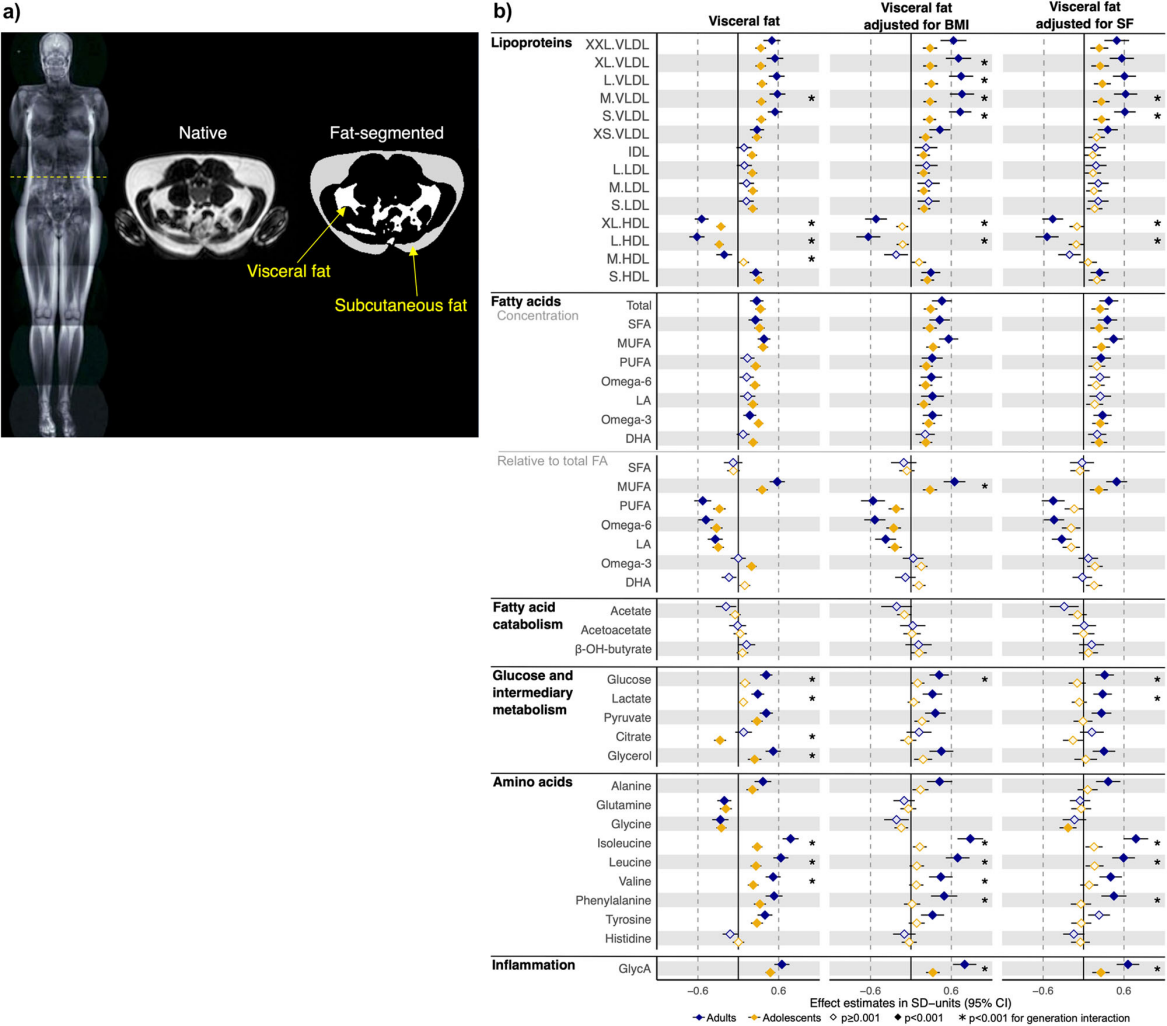
Associations between visceral fat measured directly with magnetic resonance imaging (MRI) and selected key metabolomic measures in adolescents and adults. **a** Visceral fat, which is fat around internal organs, and subcutaneous fat, which is fat outside the abdominal cavity and underneath the skin, were quantified at the level of the umbilicus with MRI; native and fat-segmented images are shown. **b** Associations between visceral fat and metabolomic measures were studied using linear regression models in up to 507 adults and 938 adolescents. All variables were inverse rank-transformed to normality and adjusted for age, sex, age-by-sex interaction, genetic relatedness, and family environment. Visceral fat was additionally adjusted for height. In further models, visceral fat was additionally adjusted for body mass index (BMI) or subcutaneous fat (SF). The effect sizes and corresponding 95% confidence intervals (CI) are in standard deviation (SD) units. The 14 lipoprotein subfractions are 6 very-low-density lipoproteins (VLDL), 1 intermediate-density lipoprotein (IDL), 3 low-density lipoproteins (LDL), and 4 high-density lipoprotein (HDL) subfractions. The lipoproteins are sorted by size from extra-extra-large (XXL) to extra-large (XL), large (L), medium (M), small (S) and extra-small (XS). SFA saturated fatty acids, MUFA mono-unsaturated fatty acids, PUFA polyunsaturated fatty acids, Omega-6 omega-6 fatty acids, LA linoleic acid, Omega-3 omega-3 fatty acids, DHA docosahexaenoic acid, and GlycA glycoprotein acetyls.

VF and subcutaneous fat (SF) were measured from T1-weighted MRI of the abdomen acquired on a Gyroscan NT 1.0-T scanner (Philips Healthcare, Best, Netherlands) in adolescents, and on an Avanto 1.5-T scanner (Siemens, Erlangen, Germany) in adults. A 10-mm-thick axial slice at the level of the umbilicus was selected to quantify VF and SF, in cubic centimeters, with a semiautomatic method described previously^14^ (Fig. 1a). BMI was calculated as body weight in kilograms (0.1-kg precision) divided by height in meters squared (0.1-cm precision).

#### Serum metabolomic profiling

Overnight fasted serum samples were analyzed using high-throughput nuclear magnetic resonance (NMR) spectroscopy (Nightingale Health Ltd, Helsinki, Finland). The platform (2016 quantification version) assesses 228 metabolic measures, including lipoproteins and lipids within 14 lipoprotein subfractions, which are 6 very-low-density lipoprotein (VLDL), 1 intermediate-density lipoprotein (IDL), 3 low-density lipoprotein (LDL), and 4 high-density lipoprotein (HDL) subfractions. The lipoprotein lipids within these subfractions include triglycerides (TGs), cholesteryl esters (CEs), free cholesterol and phospholipids. The platform also quantifies apolipoprotein B and apolipoprotein A1, as well as multiple measures of fatty acids (FAs), breakdown products of FAs, glucose and intermediary metabolism, amino acids, and a marker of inflammation, glycoprotein acetyls (GlycA). The details of the method have been described elsewhere^15,16^. Shortly, a serum sample volume of 350 μl was used for the analysis. Before the NMR measurements, the samples were mixed with a sodium phosphate buffer and moved to the NMR tubes using automated liquid handlers. The metabolomic quantifications were completed using 500 MHz and 600 MHz spectrometers (Bruker AVANCE III and Bruker AVANCE III HD) equipped with a robotic sample changer that enabled sample handling in a cooled (+6 °C) temperature. Lipoprotein and low-molecular-weight metabolites were measured from the original serum samples. Subsequently, the same samples went through a lipid extraction procedure enabling the measurements of lipid data. The downstream processing of the NMR spectra was done using automated computational algorithms, the intellectual property rights of which belong to the Nightingale Health Ltd. The output provided by the company is a list of concentrations of different metabolic measures complemented with selected ratios.

### Statistical analyses

Prior to association testing, all variables were transformed using the rank-based inverse normal transformation^17^ and adjusted for age, sex, age-by-sex interaction, genetic relatedness and shared family environment; VF and SF were additionally adjusted for height. Adjustments were done using “lmekin” function from “coxme” R package^18^, which allows inclusion of genetic relationship matrix in the model.

In primary association analyses, we tested associations of VF with 228 metabolomic measures by fitting linear models separately in adolescents and adults: in these models, each of the metabolomic measures served as an outcome and VF served as an explanatory variable. Due to the high correlation of the metabolomic measures, the number of independent tests is lower than the number of tested measures. To conduct multiple testing corrections, we carried out a principal component analysis of the metabolomic data^19^, which is useful in estimating the number of independent tests when correlated data are analyzed. We found that the first 24 and 25 principal components explained 95% of variation among the 228 metabolomic measures in adolescents and adults, respectively (Fig. S1). Accordingly, we set our statistical significance threshold at *p* < 0.001 (0.05/(24 + 25) to correct for 24 + 25 independent tests in two generations.

In secondary association analyses, we tested whether the metabolomic associations of VF remain after additional adjusting for BMI or SF. We also fitted models in a pooled sample to test for adiposity-by-age group interactions in order to explore whether the metabolomic associations of the adiposity traits differ between adolescents and adults. To study if the associations differ by sex, we also fitted the regression models in sex-specific subsamples—these models were adjusted for age, height, genetic relatedness, and shared family environment.

Finally, we tested—in both adolescents and adults—if the BMI-adjusted metabolomic profile of VF is similar to the BMI-adjusted metabolomic profiles of T2D, MI and IS from previous open-access studies of adults using the same Nightingale NMR-based metabolomics platform we used in the present study^6,7^. Data for 87 metabolomic variables that were available in all studies were used to determine correlations between the effect estimates of VF and the effects estimates derived from odds ratios of T2D, MI and IS. First, odds ratios (and corresponding 95% confidence intervals (CIs)) were log-transformed to get all these values on the beta estimate scale and, subsequently, each beta (and CI) was scaled to the effect on VLDL-TG to facilitate comparison of the effect similarities regardless of the magnitude of the absolute effect that may vary due to differences in study settings; VLDL-TG was selected to be the scaling factor because it is among the metabolic measures most strongly associated with VF and it shows a positive association also with T2D, MI, and IS. These scaled beta estimates were used to determine correlations (Pearson’s *r*) between the metabolomic profiles of VF vs. T2D, MI and IS. We further derived standard errors for each association as follows: SE = (CI95upper–CI95lower)/(2*1.96). Using the scaled beta estimates and derived standard errors, we estimated for each metabolic measure if the difference between the effects of VF vs. CMD (T2D, MI, or SI) is significant: *Z* = (beta_1_–beta_2_)/sqrt(se_1_^2 + se_2_^2). The *p* values for *Z* were derived from the normal distribution. We further re-evaluated the similarities between metabolomic profiles of VF and CMD in sensitivity analyses using a subset 15 of noncorrelated metabolic measures identified using hierarchical clustering (R function “hcut”).

### Reporting summary

Further information on research design is available in the Nature Research Reporting Summary linked to this article.

## Results and discussion

In the studied sample, the prevalence of overweight or obesity was 68% in adults and 28% in adolescents, which is similar to the Canadian population at large^20,21^. The average BMI was 27.9 kg/m^2^ in adults and 21.8 kg/m^2^ in adolescents. Consistent with previous research indicating that VF increases with age more than SF^11^, VF volume was four-times larger and SF volume two-times larger in adults than in adolescents. On average, both adults and adolescents were normoglycemic and normolipidemic; as expected, glucose, total TGs, LDL-cholesterol and HDL-cholesterol were higher in adults than in adolescents (Table 1).

**Table 1:** Basic characteristics of studied participants.

Characteristic	Adolescents (*n* = 938)	Adults (*n* = 507)	*p*_difference_
Male, %	47.5	44.6	0.3
Age, years	14.6 ± 1.8	49.0 ± 4.8	*p* < 2e−308
Height, cm	163.2 ± 9.6	167.1 ± 8.5	6.8e−24
Weight, kg	58.7 ± 15.1	78.1 ± 16.7	5.4e−110
BMI, kg/m^2^	21.8 ± 4.5	27.9 ± 5.4	1.4e−114
Overweight or obese^a^, %	28.1	68.2	7.7e−49
Visceral Fat, cm^3^	22.1 ± 19.1	83.0 ± 60.3	4.4e−159
Subcutaneous fat, cm^3^	126.1 ± 102.0	283.9 ± 148.3	2.7e−130
Total TG, mmol/l	0.9 ± 0.4	1.4 ± 0.7	3.0e−57
LDL-C^b^, mmol/l	1.2 ± 0.4	1.6 ± 0.5	1.9e−79
HDL-C, mmol/l	1.3 ± 0.3	1.4 ± 0.4	5.7e−4
Glucose, mmol/l	3.6 ± 0.3	4.1 ± 0.8	3.9e−112

Proportions (%) or means ± standard deviations are shown.

^a^In adults, “overweight or obese” refers to BMI ≥ 25 kg/m^2^ and, in adolescents, the threshold was set to BMI ≥ 1 SD (equivalent to BMI ≥ 25 kg/m^2^ at 19 years) determined using the WHO BMI *Z*-score calculator, which takes into account age, sex, height and weight^21^.

^b^Levels of NMR-based LDL-C are lower than the LDL-C levels measured with common assays.

**Fig. 2:**
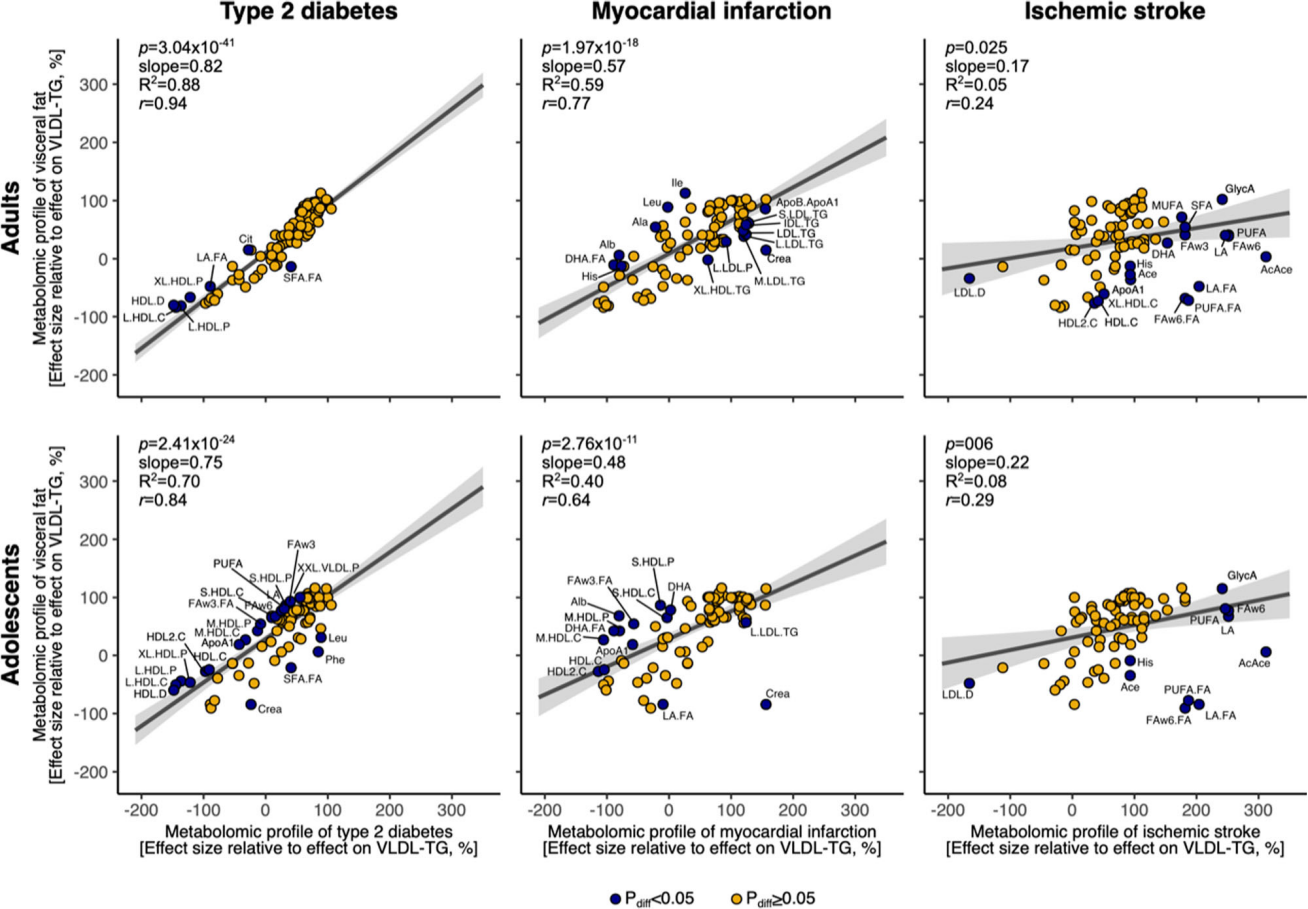
Correlation of the body mass index (BMI)-adjusted metabolomic profile of visceral fat with the BMI-adjusted metabolomic profiles of type 2 diabetes, myocardial infarction and ischemic stroke in adults and adolescents. Effect estimates of the metabolomic associations of type 2 diabetes, myocardial infarction, and ischemic stroke were extracted from open-access studies that employed the same nuclear magnetic resonance (NMR)-based metabolomics platform we used here^6,7^. The correlations are plotted using 87 metabolomic measures publicly available in all three studies. The study-specific effect sizes are scaled with respect to the effect size on very-low-density triglyceride (VLDL-TG) concentration to facilitate comparison of effect sizes originating from different study settings. Circles in dark blue indicate the metabolomic measures that showed significantly different effects of VF vs. type 2 diabetes, myocardial infarction, or ischemic stroke, and the shaded area shows the 95% confidence interval for the line indicating the linear fit between the metabolomic profiles. Ace acetate, AcAce acetoacetate, Ala alanine, Alb albumin, ApoA1 apolipoprotein A-I, ApoB apolipoprotein B, ApoB.ApoA1 ratio of ApoB to ApoA1, Cit citrate, Crea creatinine, GlycA glycoprotein acetyls, His histidine, Ile isoleucine, LA linoleic acid, Leu leucine, Phe phenylalanine, FAw3 omega-3 fatty acids, FAw6 omega-6 fatty acids, and, for fatty acids, abbreviations ending “.FA” indicate fatty acid proportions relative to total fatty acids. The 14 lipoprotein subfractions are 6 very-low-density lipoprotein (VLDL), 1 intermediate-density lipoprotein (IDL), 3 low-density lipoprotein (LDL), and 4 high-density lipoprotein (HDL) subfractions. The lipoproteins are sorted by size from extra-extra-large (XXL) to extra-large (XL), large (L), medium (M), small (S) and extra-small (XS). For lipoprotein particles, P particle concentration and D particle size.

The key results are shown in Figs. 1 and 2. Serum levels of all 228 metabolomic measures are given in Supplementary Data 1. Inter-generational differences in the metabolomic measures were similar to those described previously^22^ (Supplementary Data 1). The full association profile of VF with the 228 metabolomic measures is plotted in Fig. S2 and a numerical presentation is given in Supplementary Data 2. The complete results from the remaining BMI- and SF-adjusted models, as well as sex-separate models, are plotted in Figs. S3–S8 and tabulated in Supplementary Data 3–8. Below, we highlight the main findings.

### Many aspects of the metabolic profile of VF are in line with the direct effects of VF on the liver

We found that higher VF is associated—independently of BMI or SF—with higher TG-rich VLDL particles, lower HDL particles in the very large and large subfractions, higher HDL particles in the small subfraction, higher branched-chain amino acids (BCAAs) and higher systemic inflammation (Fig. 1b). Although directionality cannot be interpreted from these cross-sectional associations, previous evidence strongly suggests that obesity-related aberrations in circulating metabolome are likely a consequence rather than a cause of excess adiposity^4,10^. Many of the observed associations speak for the direct effect of VF on the liver: VF unlike SF drains to the liver via the portal circulation, thus exposing the liver directly to the VF-released proinflammatory molecules and FAs, among others^11,23^. Many of these molecules (e.g., tumor necrosis factor α^24^) impair hepatic actions of insulin and, thus, increase hepatic gluconeogenesis and synthesis of TG-rich VLDL particles^25^, and decrease hepatic catabolism of AAs (and BCAAs in particular). Higher concentrations of TG-rich VLDL particles in the systemic circulation stimulate CE-transfer protein to transfer TGs from VLDL to HDL particles in exchange for CEs^26^, producing TG-rich HDL particles that are smaller and more prone to elimination by the kidneys, thus lowering the circulating pool of HDL particles, an effect that may compromise removal of atherogenic cholesterol from the circulation (i.e., via HDL-mediated reverse cholesterol transport)^27^. Finally, VF-released proinflammatory cytokines (e.g., interleukin 6) stimulate liver synthesis and release of inflammatory molecules, such as C-reactive protein and GlycA, promoting inflammation and atherogenesis in the systemic circulation, among others^28^. Thus, VF appears to be closely related to hepatic insulin resistance and related glucose and lipid abnormalities, as well as systemic inflammation; these processes in turn are the key pathobiological processes of T2D and atherosclerotic vascular disease, such as MI.

### Many of the VF-associated metabolic variations were significantly different between the two age groups

The BMI or SF-adjusted VF associations with higher TG-rich VLDL particles and inflammation were present in both generations, but the effect estimates were significantly larger in adults than in adolescents (Fig. 1b). Further, in the BMI or SF-adjusted models, VF associations with higher glucose and intermediary metabolites and BCAAs remained significant in adults only with significant metabolite-by-age group interactions. A probable factor contributing to these age group-specific metabolic associations of VF may be the presence of fatty liver: VF accumulation is closely correlated with fatty liver^29^ and fatty liver is a key modulator of levels of these metabolites^30,31^. Importantly, the prevalence of fatty liver increases with age, affecting an estimated ~13% of adolescents aged 12–17 years^32^ and ~46% of working-age adults^33^. Other theoretical contributors to the age group differences include the longer exposure to the adverse secretory effects of VF in adults than in adolescents (due to older age), which may lead to more severe health consequences of obesity^34^, or the differences in the biology of VF expansion, which involve mostly adipocyte hypertrophy in adults and adipocyte proliferation in adolescents (larger adipocytes exhibit more adverse secretary profile^35^). Lastly, VF was associated with higher levels of cholesterol-rich IDL and LDL particles in adolescents but not in adults. While this difference may be at least partially due to the larger sample size in adolescents than in adults, biological factors—such as growth hormone, which peaks in adolescence^36^, is inhibited by higher adiposity^37^, and modulates the number of LDL receptors^38^—may play a role. As IDL and LDL particles are atherogenic^39^, excess VF may promote atherosclerosis already in youth^2^. Consistently, children and adolescents with a genetic growth hormone deficiency have higher levels of LDL-cholesterol, higher thickness of the carotid intima media, and higher mortality rates from cardiovascular disease^40^. Of note, we did not find significant sex differences in either age group (Figs. S7 and S8).

### Similar metabolic pathways may be involved in VF, T2D, and MI, but less so in IS

We found that, in both generations, the above-described BMI-adjusted metabolomic profile of VF was highly similar to those of T2D (adults: *R*^2^ = 0.88; adolescents: *R*^2^ = 0.70) and MI (adults: *R*^2^ = 0.59; adolescents: *R*^2^ = 0.40) (Figs. 2 and S9). Results obtained in a sensitivity analysis using a subset of 15 noncorrelated metabolomic measures provided similar results (Fig. S10). Our results are consistent with a large-scale genetic study suggesting that higher VF is causally related to T2D and MI in adults^41^. Previous literature also suggests that metabolic changes typically precede the diagnosis of CMD^42^ and fatty liver^31^; our findings provide evidence that the CMD-related metabolic changes may start already at a very young age, as the metabolic signature associated with CMD in adulthood can be detected in adolescents with higher VF (Fig. 2). Further, we discovered that the BMI-adjusted metabolomic profile of VF showed lower similarity with the metabolomic profile of IS compared with MI (adults: *R*^2^ = 0.05; adolescents: *R*^2^ = 0.08). The specific reasons are not clear. Nevertheless, atherosclerosis of intracranial vs. coronary arteries exhibits several differences, some of which include lower lipid infiltration and higher fibrosis^43^. In this context, it is of note that the major differences between the metabolic profiles of IS and VF (and not between the profiles of MI and VF profile) were (1) a weaker negative association of IS with cholesterol content in the larger HDL subfractions, and (2) stronger positive associations of IS with polyunsaturated-FA-to-total-FA ratios and acetoacetate, a breakdown product of FAs. The latter is interesting, as elevated circulating polyunsaturated FAs have recently been causally related to aortic stenosis^44^, which is a progressive fibrotic disease of aortic valves.

### VF explains a sizable proportion of variance in metabolomic measures

For example, isoleucine is a BCAA demonstrating one of the largest effect sizes in adults but not adolescents: in adults, VF explained 23%, 13%, and 17% of variance in the isoleucine level in the basic, BMI- and SF-adjusted models, respectively, whereas, in adolescents, the corresponding variances explained were 6%, 1%, and 2%. In comparison, age explained only 2% of variance in isoleucine levels in all three models in adults and 0% variance in all three models in adolescents. The inflammatory marker GlycA demonstrated one of the largest effect sizes in both adults and adolescents: in adults, VF explained 22%, 12%, and 11% of variance in GlycA levels in the basic, BMI- and SF-adjusted models, respectively, and, in adolescents, the respective values were 17%, 6%, and 1%. In comparison, age explained only 2% of variance in GlycA levels in all three models and in both adults and adolescents.

### Strengths and limitations

Unlike previous studies, we measured body adiposity directly with MRI and, therefore, could also assess associations of VF independently of SF. This is important, as VF more than other fat in the body increases risk for CMD^8^. VF can only be measured with MRI or computerized tomography; other methods only estimate VF indirectly (e.g., dual-energy X-ray absorptiometry)^8^. Further, unlike previous studies, we analyzed data from two generations, thus providing a broader lifespan perspective to the VF-associated metabolic aberrations. Our study, however, is observational and as such cannot make causal conclusions. Also, it would be highly beneficial to assess the impact of fatty liver in these associations; this was not possible in this report due to the lack of relevant data. Given that we studied here adults and adolescents from a single population, replication of our findings in other populations and ethnicities would be of high value.

## Conclusions

In conclusion, this is the first study to investigate associations of MRI-quantified VF with circulating metabolomic measures in two generations—adolescents and their middle-aged parents. In both generations, VF accumulation was associated with major aberrations in TG and FA metabolism and systemic inflammation. In adults only, VF accumulation was additionally associated with major aberrations of glucose and amino acid metabolism. Most of these associations remained after adjusting for BMI or SF, which indicates that visceral adiposity independent of general adiposity may be detrimental for cardiometabolic health. Consistently, the BMI-adjusted metabolomic profile of VF is closely associated with the metabolomic aberrations increasing risk for T2D and ischemic heart disease not only in adults but also in adolescents.

## Supplementary information

Supplementary Data

Description of Additional Supplementary Files

Supplementary Information

Reporting Summary

## Data Availability

To protect the privacy of the Saguenay Youth Study participants, individual level data are stored on a private server and only available upon request from the Saguenay Youth Study (zdenka.pausova@sickkids.ca). All summary statistics are available within the related Supplementary Files. Source data for the figures can be found in Supplementary Data 2–4. The previously published metabolomic association profiles of cardiometabolic diseases are available within the Supplementary Materials of the papers by Ahola-Olli et al. and Holmes et al.

## Code availability

All analyses were conducted using R libraries described in Methods and, thus, this paper does not report original code. The exact R commands used are available upon request.
