# Supplementary material for: Visceral adiposity is associated with metabolic profiles predictive of type 2 diabetes and myocardial infarction
Source: Commun Med (Lond). 2022 Jul 1;2:81. doi: 10.1038/s43856-022-00140-5 (PMC9249739; doi:10.1038/s43856-022-00140-5)
Supplement: Supplementary file 2 — Description of Additional Supplementary Files [file 43856_2022_140_MOESM2_ESM.pdf]

Description of Additional Supplementary Files

File Name: Supplementary Data

Description: Source data for the main figures
