# Supplementary material for: Visceral adiposity is associated with metabolic profiles predictive of type 2 diabetes and myocardial infarction
Source: Commun Med (Lond). 2022 Jul 1;2:81. doi: 10.1038/s43856-022-00140-5 (PMC9249739; doi:10.1038/s43856-022-00140-5)
Supplement: Supplementary file 3 — Supplementary Information [file 43856_2022_140_MOESM3_ESM.pdf]

Supplementary Figures

Visceral adiposity is associated with metabolic profiles predictive of type 2 diabetes and myocardial infarction

Javeria Raheem, Eeva Sliz, Jean Shin, Michael V. Holmes, G. Bruce Pike, Louis Richer, Daniel Gaudet, Tomas Paus, Zdenka Pausova

Table of Contents

Figure S1. Cumulative variance explaining the variation in the metabolomics data in SYS adolescents and adults. 1

Figure S2. Associations of visceral fat with 228 metabolic measures in adolescents and adults. 2

Figure S3. Associations of visceral fat adjusted for BMI with 228 metabolic measures in adolescents and adults. 3

Figure S4. Associations of visceral fat adjusted for subcutaneous fat with 228 metabolic measures in adolescents and adults. 4

Figure S5. Associations of BMI with 228 metabolic measures in adolescents and adults. 5

Figure S6. Associations of subcutaneous fat with 228 metabolic measures in adolescents and adults. 6

Figure S7. Associations of visceral fat with 228 metabolic measures in adolescent females and males. 7

Figure S8. Associations of visceral fat with 228 metabolic measures in adult females and males. 8

Figure S9. Metabolomic profiles of visceral fat, type 2 diabetes, myocardial infarction and stroke. 9

Figure S10. Correlations of the metabolic profiles of visceral fat and the ones of type 2 diabetes, myocardial infarction, and ischemic stroke using a subset of noncorrelated 15 metabolic measures. 10

References 11

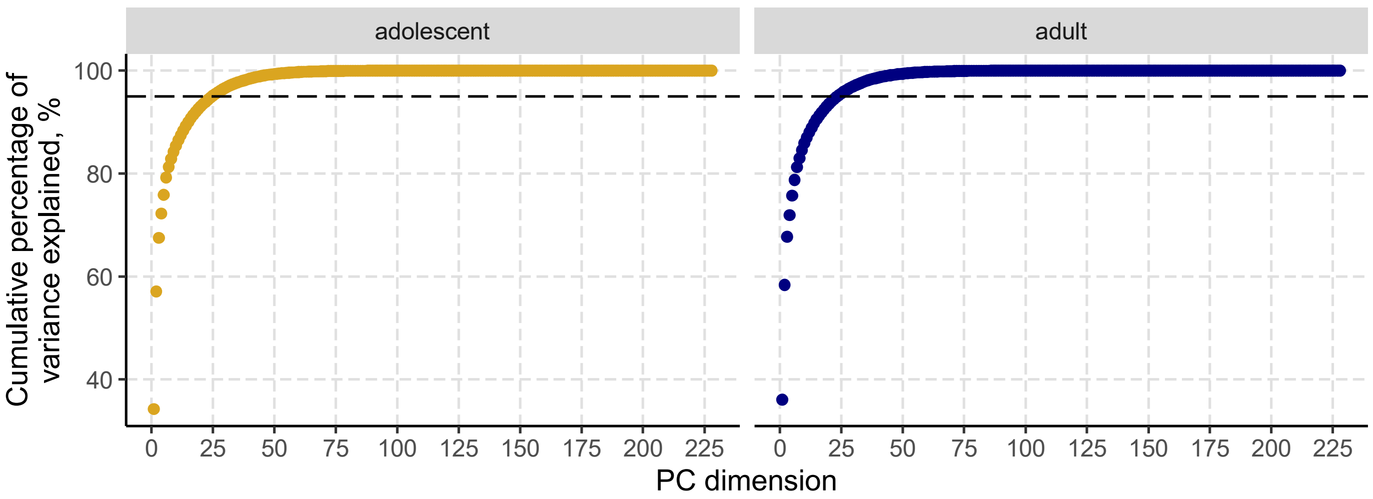

Figure S1. Cumulative variance explaining the variation in the metabolomics data in SYS adolescents and adults. The black dashed line indicates the threshold for 95%.

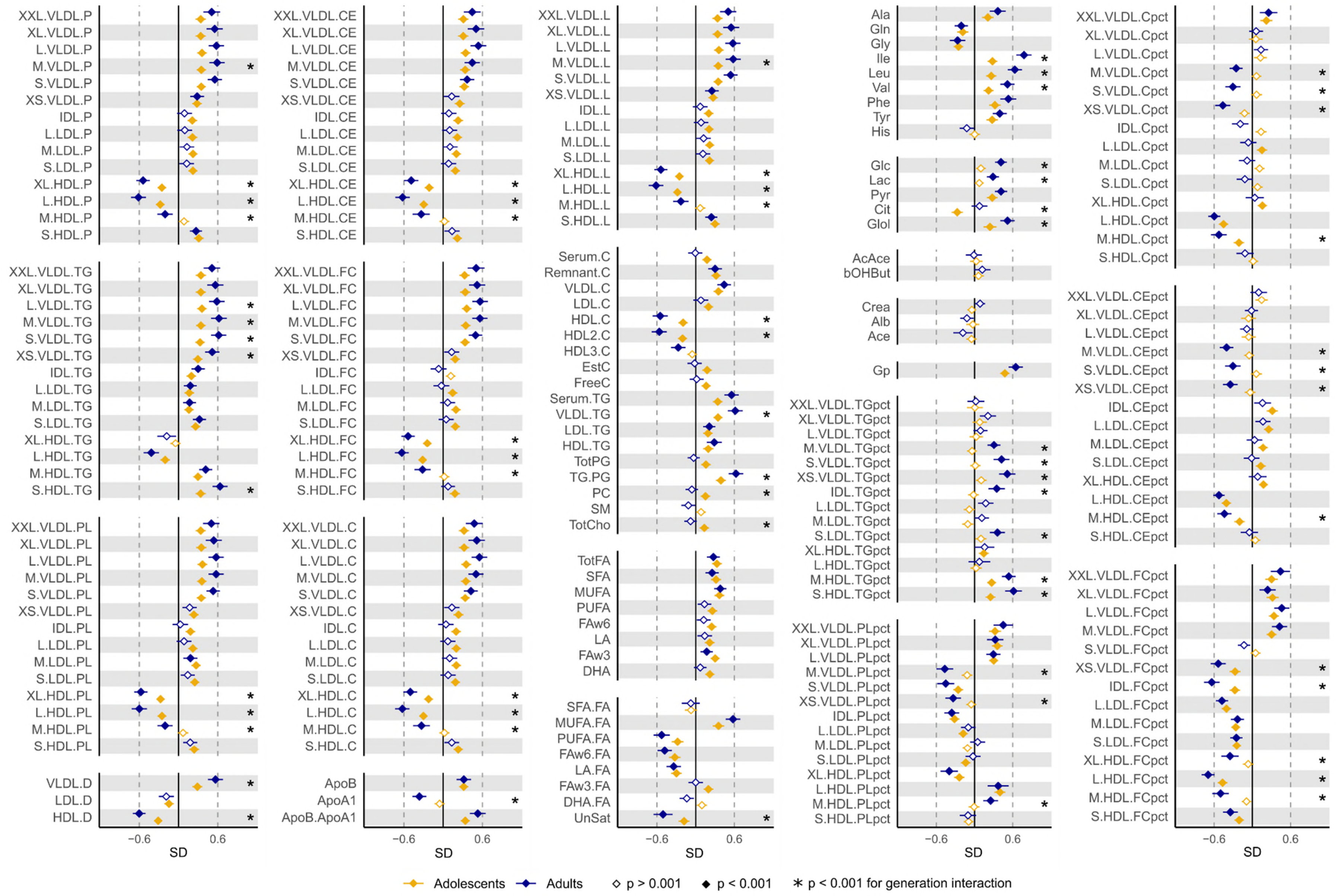

Figure S2. Associations of visceral fat with 228 metabolic measures in adolescents and adults.

Prior to analyses, all variables were inverse rank-transformed to normality and adjusted for age, sex, age-by-sex interaction, genetic relatedness, and family environment. VF was additionally adjusted for height. The linear models were fitted in adolescents (yellow) and adults (blue) separately and in a pooled sample for investigating VF-by-age group interactions. Fill indicates significant associations at $p<0.001$ and asterisks denote significant ($p<0.001$) VF-by-age group interactions. The effect sizes and corresponding 95% confidence intervals are in standard deviation (SD) units.

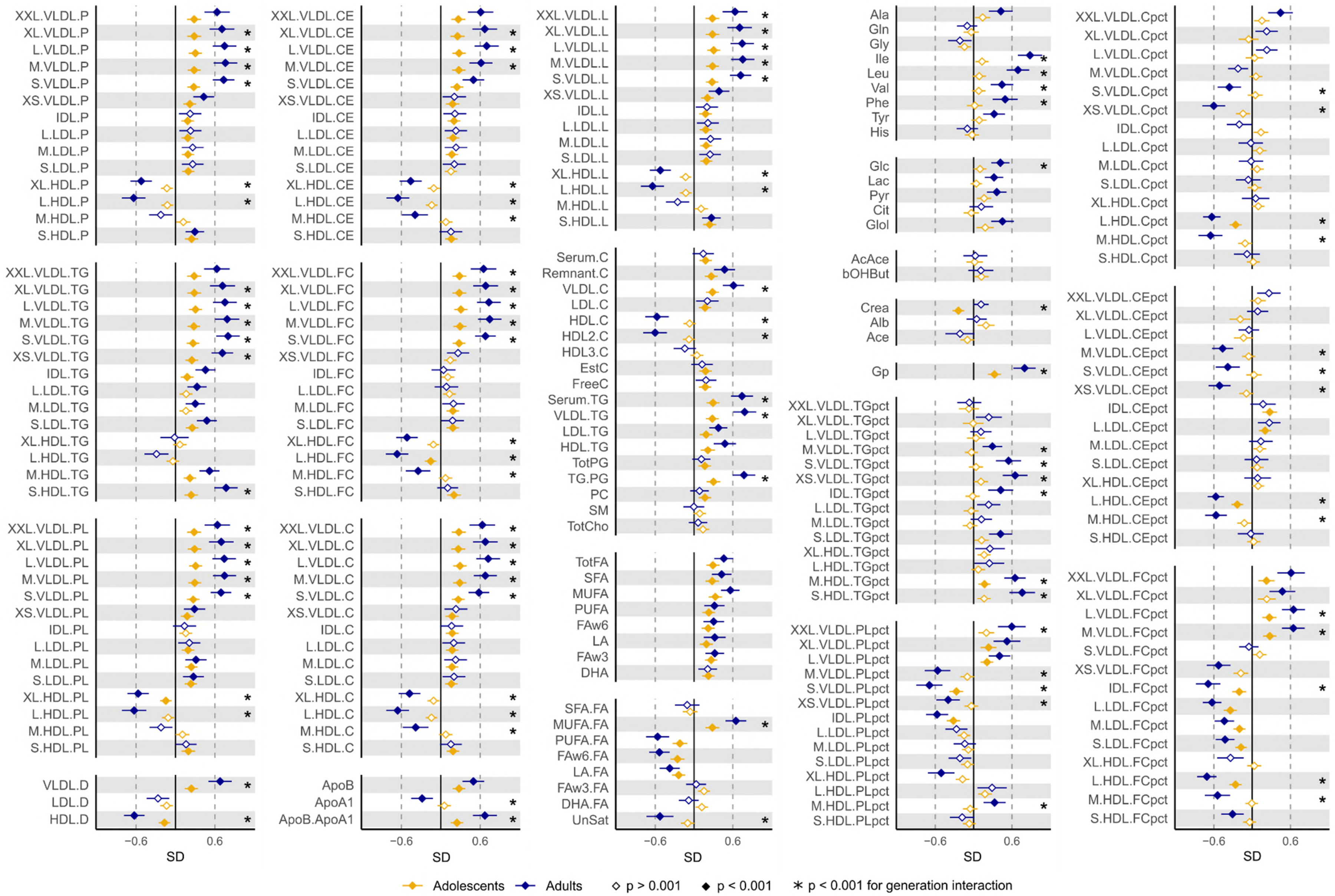

Figure S3. Associations of visceral fat adjusted for BMI with 228 metabolic measures in adolescents and adults.

Prior to analyses, all variables were inverse rank-transformed to normality. Metabolic measures and VF were adjusted for age, sex, age-by-sex interaction, genetic relatedness, and family environment. VF was additionally adjusted for height and BMI. The linear models were fitted in adolescents (yellow) and adults (blue) separately and in a pooled sample for investigating VF-by-age group interactions. Fill indicates significant associations at p<0.001 and asterisks denote significant (p<0.001) VF-by-age group interactions. The effect sizes and corresponding 95% confidence intervals are in standard deviation (SD) units.

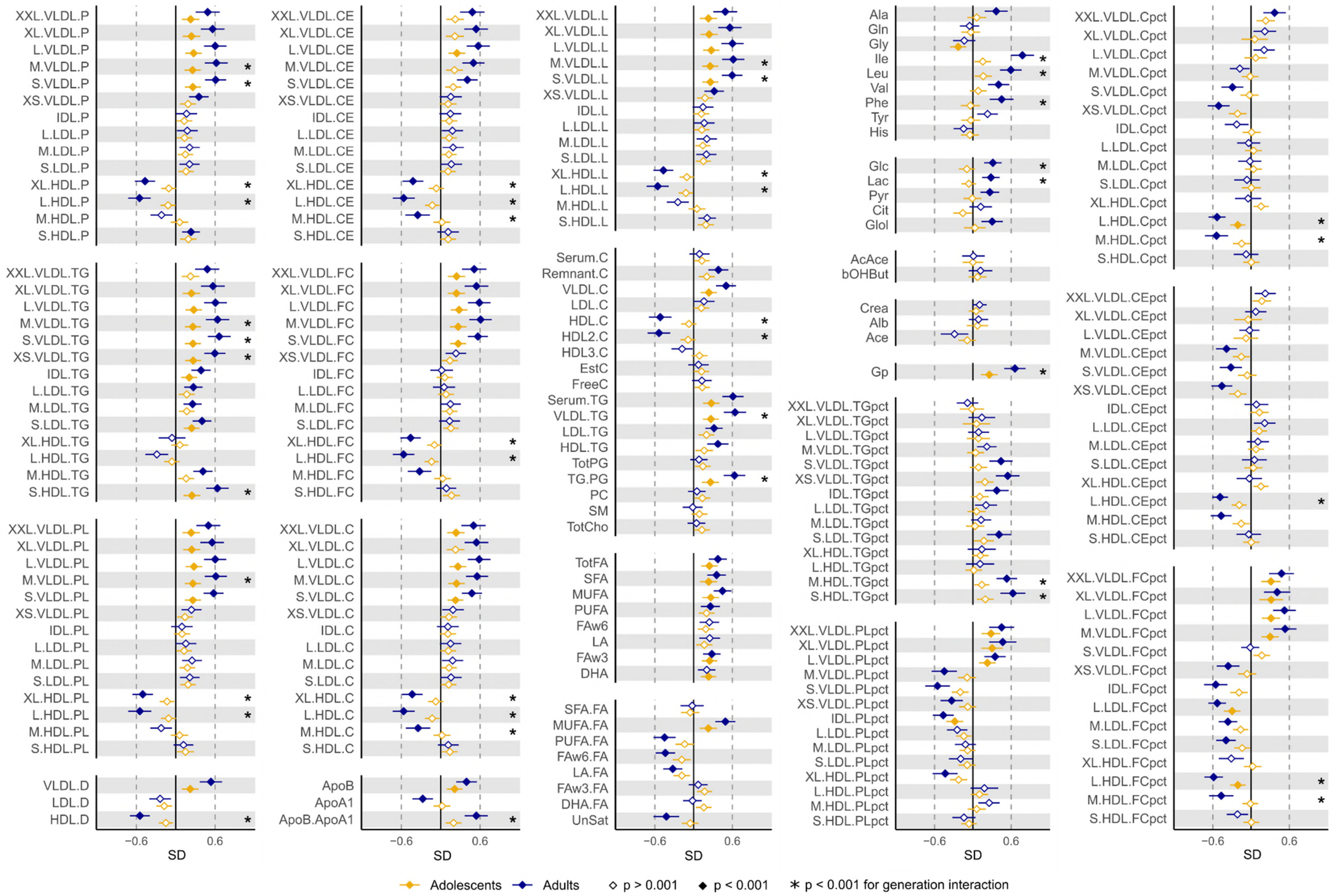

Figure S4. Associations of visceral fat adjusted for subcutaneous fat with 228 metabolic measures in adolescents and adults.
Prior to analyses, all variables were inverse rank-transformed to normality. Metabolic measures and VF were adjusted for age, sex, age-by-sex interaction, genetic relatedness, and family environment. VF was additionally adjusted for height and SF. The linear models were fitted in adolescents (yellow) and adults (blue) separately and in a pooled sample for investigating VF-by-age group interactions. Fill indicates significant associations at $p<0.001$ and asterisks denote significant ($p<0.001$) VF-by-age group interactions. The effect sizes and corresponding 95% confidence intervals are in standard deviation (SD) units.

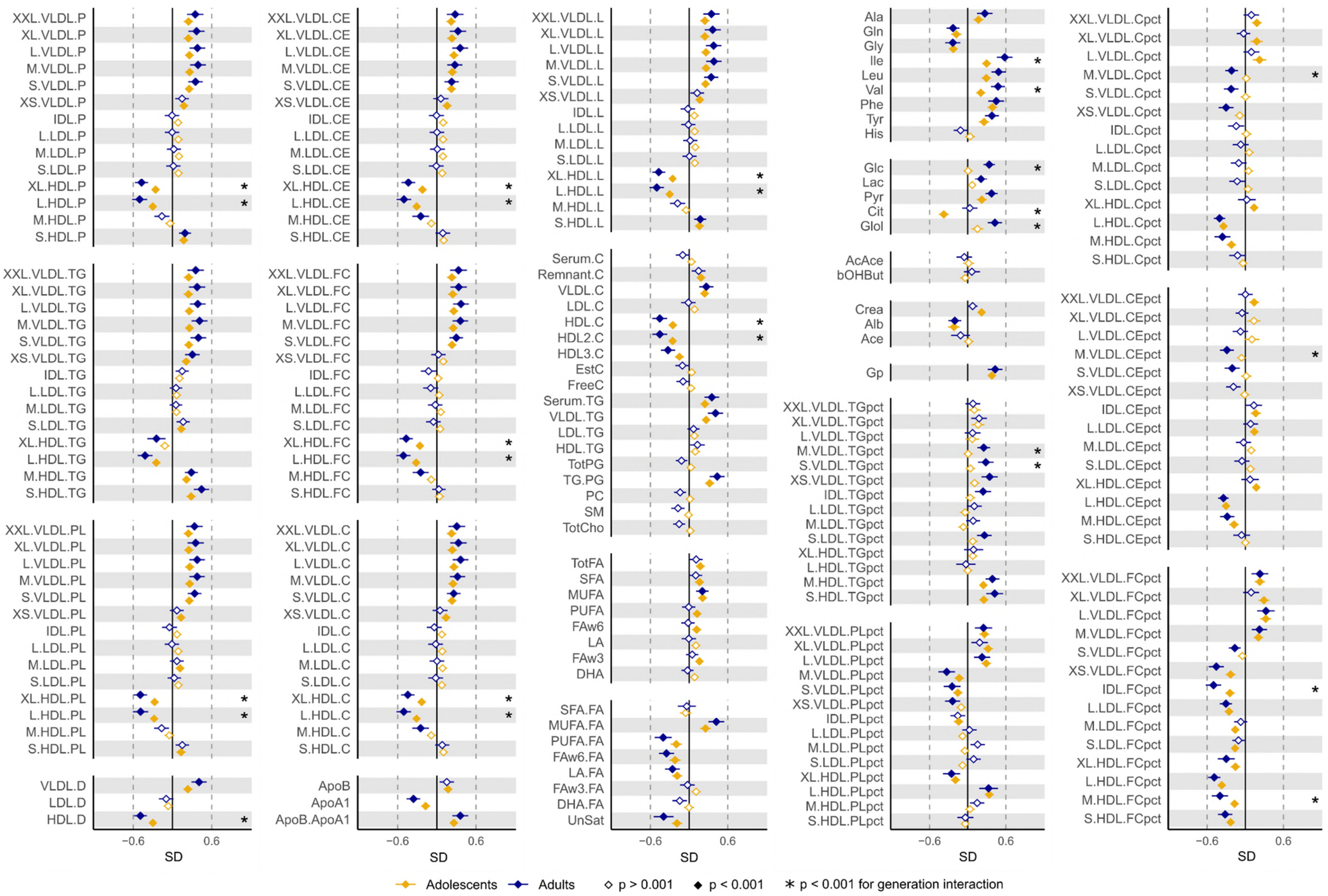

Figure S5. Associations of BMI with 228 metabolic measures in adolescents and adults.

Prior to analyses, all variables were inverse rank-transformed to normality and adjusted for age, sex, age-by-sex interaction, genetic relatedness, and family environment. BMI was additionally adjusted for height. The linear models were fitted in adolescents (yellow) and adults (blue) separately and in a pooled sample for investigating BMI-by-age group interactions. Fill indicates significant associations at $p<0.001$ and asterisks denote significant ($p<0.001$) BMI-by-age group interactions. The effect sizes and corresponding 95% confidence intervals are in standard deviation (SD) units.

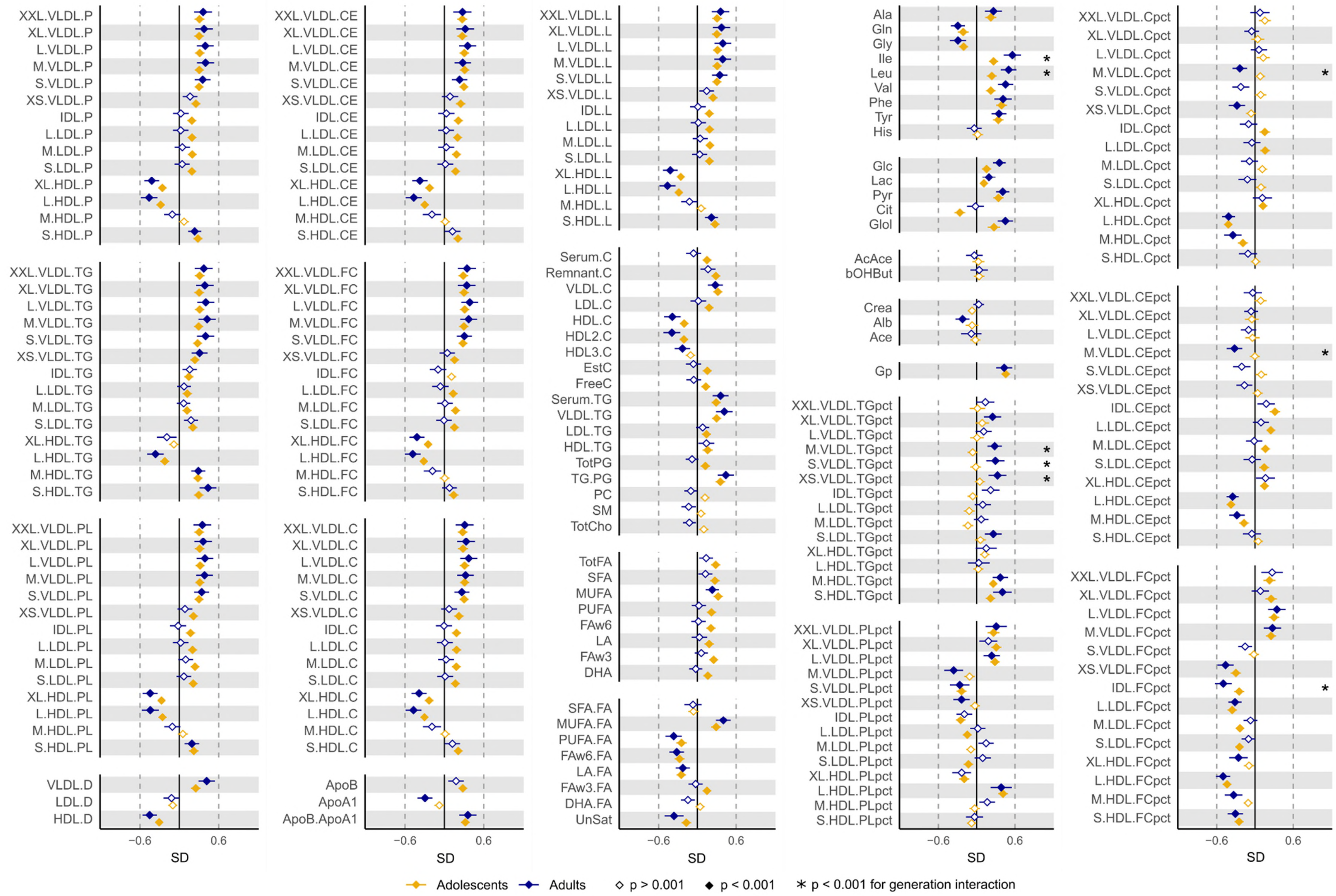

Figure S6. Associations of subcutaneous fat with 228 metabolic measures in adolescents and adults.

Prior to analyses, all variables were inverse rank-transformed to normality and adjusted for age, sex, age-by-sex interaction, genetic relatedness, and family environment. SF was additionally adjusted for height. The linear models were fitted in adolescents (yellow) and adults (blue) separately and in a pooled sample for investigating SF-by-age group interactions. Fill indicates significant associations at $p<0.001$ and asterisks denote significant ($p<0.001$) SF-by-age group interactions. The effect sizes and corresponding 95% confidence intervals are in standard deviation (SD) units.

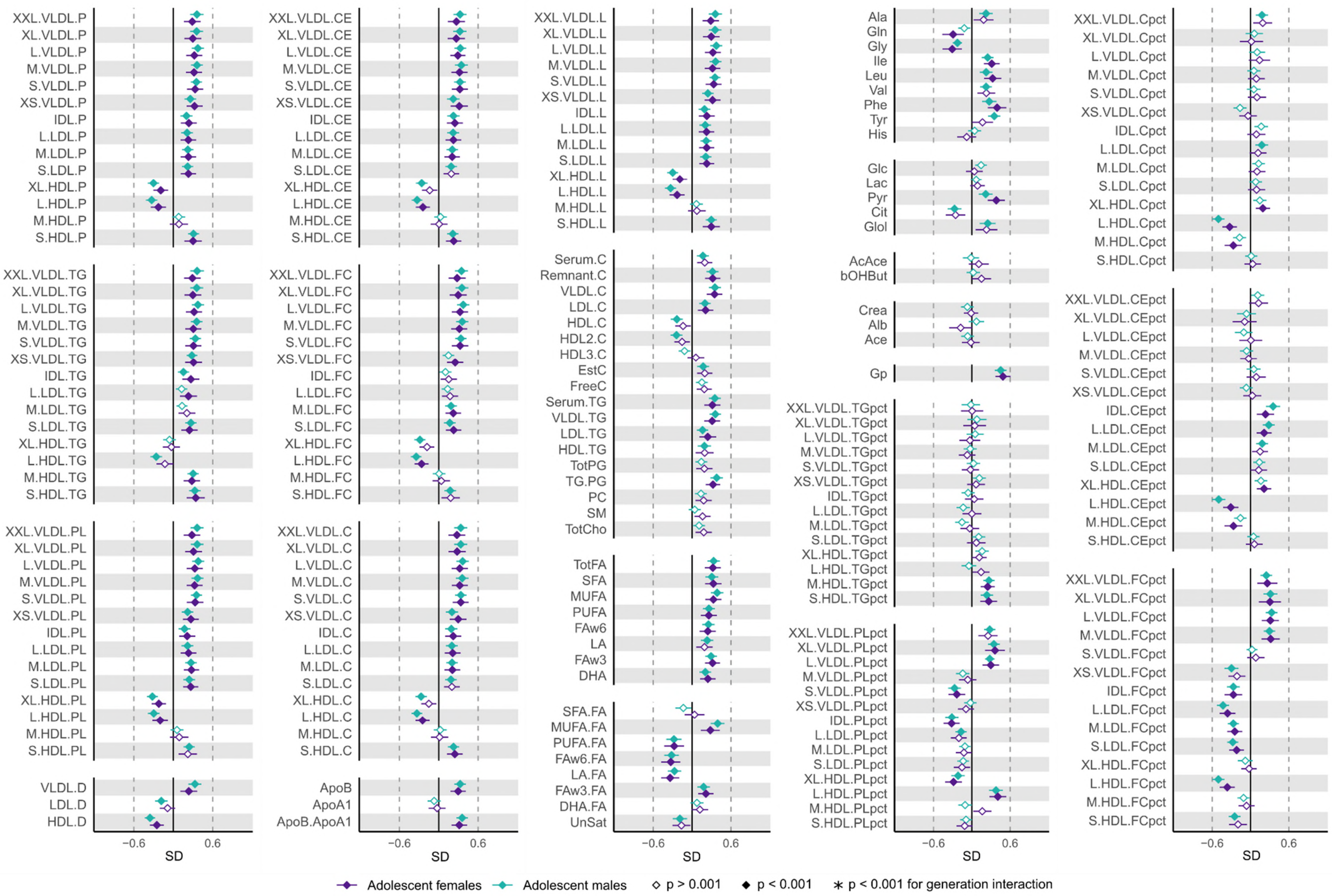

Figure S7. Associations of visceral fat with 228 metabolic measures in adolescent females and males.
Prior to analyses, all variables were inverse rank-transformed to normality and adjusted for age, genetic relatedness, and family environment. VF was additionally adjusted for height. The linear models were fitted in adolescent females (purple) and adolescent males (sea green) separately and in a pooled sample for investigating VF-by-sex interactions. Fill indicates significant associations at $p<0.001$ and asterisks denote significant ($p<0.001$) VF-by-sex interactions. The effect sizes and corresponding 95% confidence intervals are in standard deviation (SD) units.

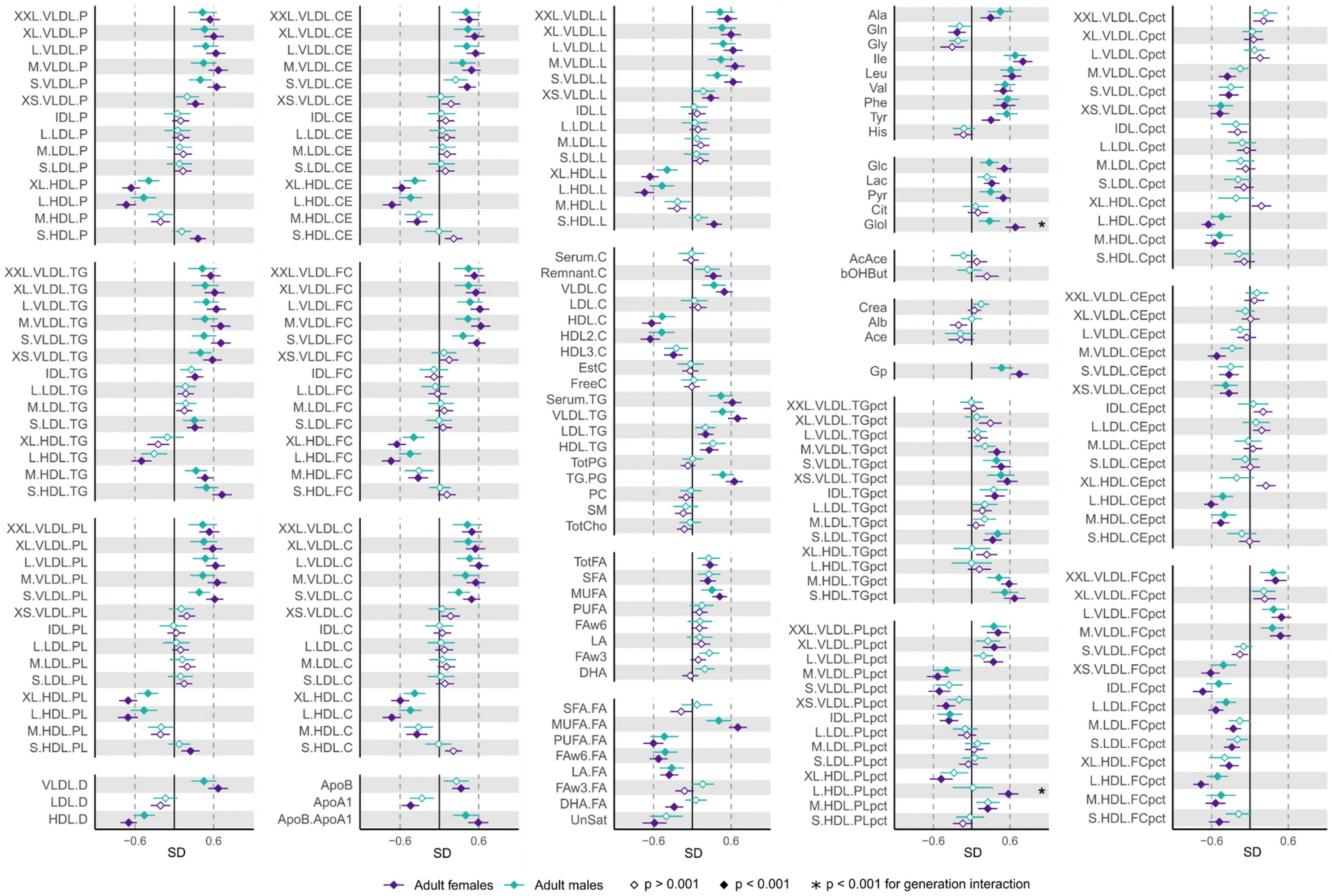

Figure S8. Associations of visceral fat with 228 metabolic measures in adult females and males.

Prior to analyses, all variables were inverse rank-transformed to normality and adjusted for age, genetic relatedness, and family environment. VF was additionally adjusted for height. The linear models were fitted in adult females (purple) and adult males (sea green) separately and in a pooled sample for investigating VF-by-sex interactions. Fill indicates significant associations at $p<0.001$ and asterisks denote significant ($p<0.001$) VF-by-sex interactions. The effect sizes and corresponding 95% confidence intervals are in standard deviation (SD) units.

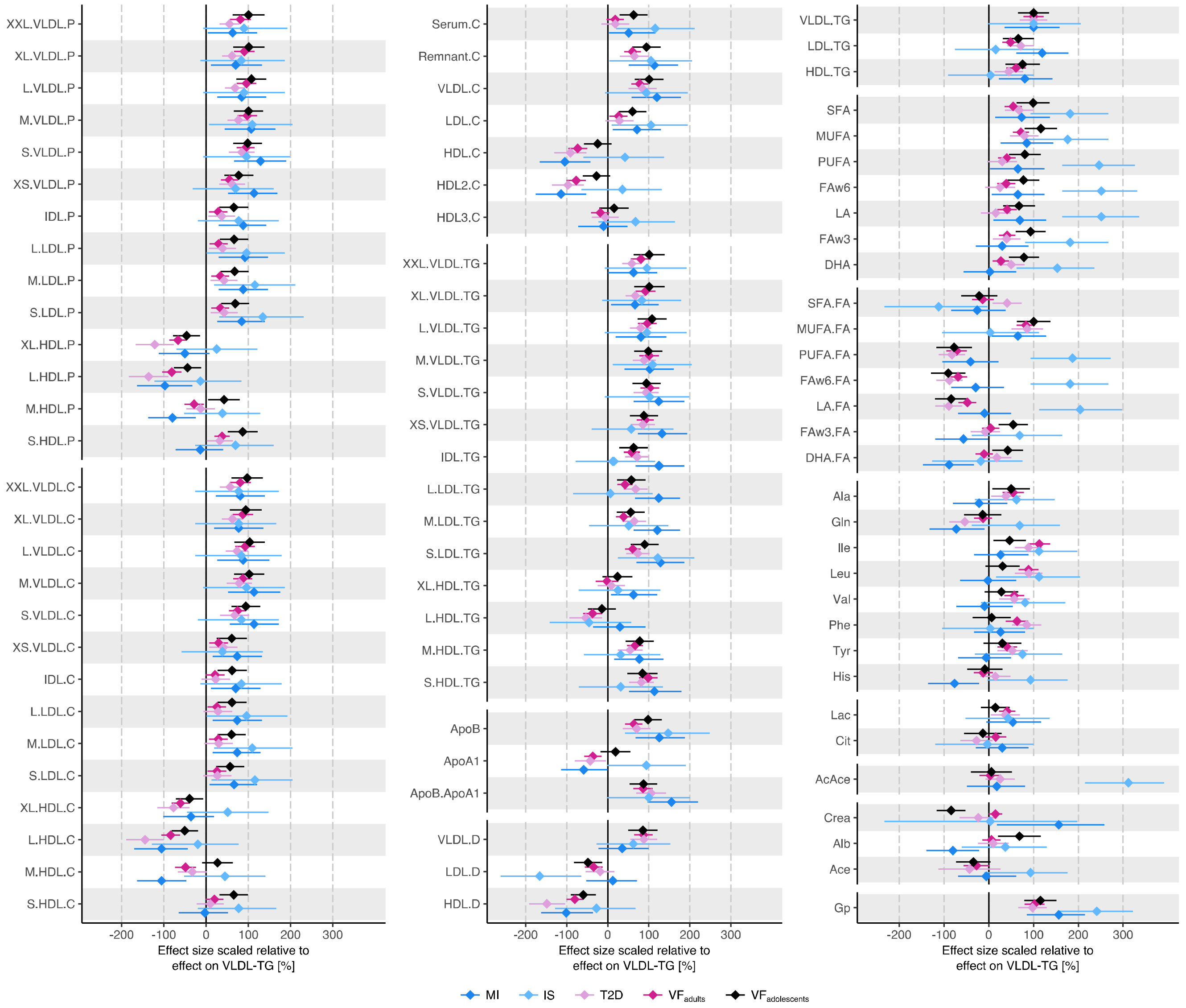

Figure S9. Metabolomic profiles of visceral fat, type 2 diabetes, myocardial infarction and stroke.
Metabolomic associations of visceral fat were determined in 507 middle-aged adults using models where each of the metabolic measures served as outcome and visceral fat as an explanatory variable. Prior to model fitting, all traits were transformed using rank-based inverse normal transformation and adjusted for age, sex, age-by-sex interaction, genetic relatedness, shared family environment, and BMI. Visceral fat was additionally adjusted for height. BMI-adjusted metabolomic profiles of type 2 diabetes, myocardial infarction and stroke were extracted from open-access sources[1,2]. The reported odds ratios were first converted to effect sizes using natural logarithms, and subsequently all the effect sizes were scaled with respect to the phenotype-specific effect size with serum total triglycerides.

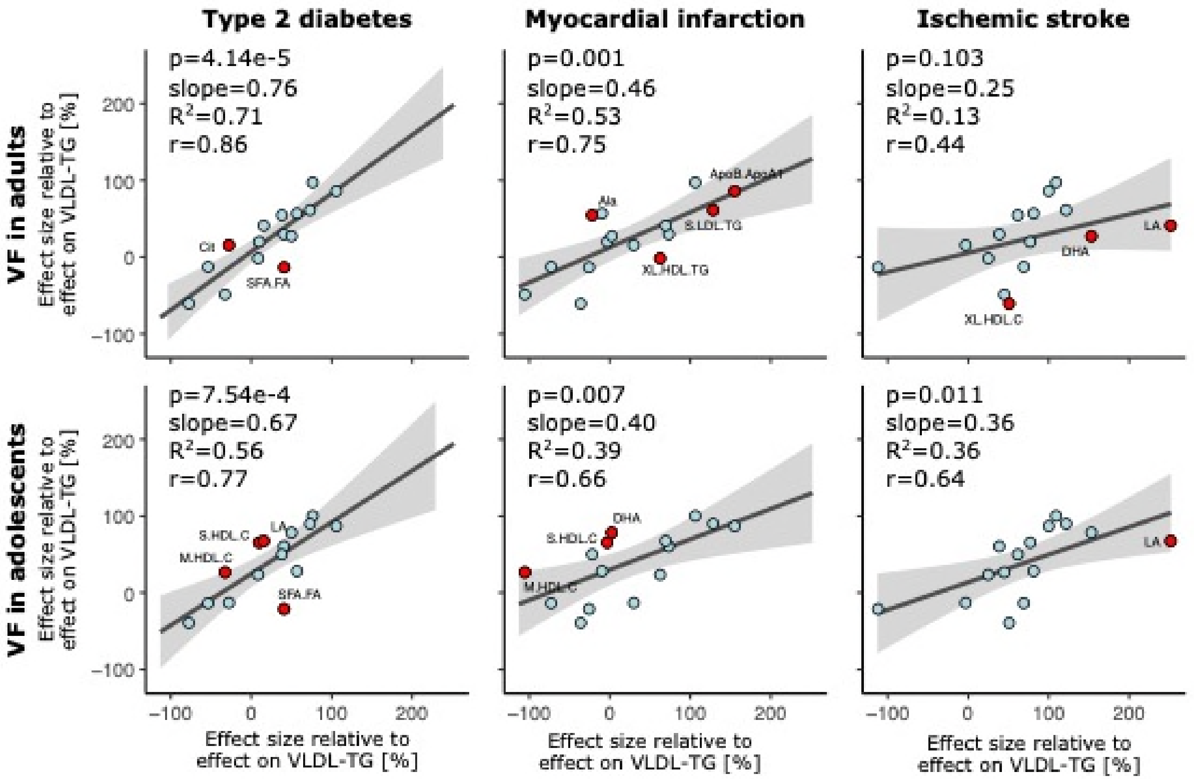

Figure S10. Correlations of the metabolic profiles of visceral fat and the ones of type 2 diabetes, myocardial infarction, and ischemic stroke using a subset of noncorrelated 15 metabolic measures.
Hierarchical clustering of the 228 metabolomic measures available in SYS was completed in R using the 'hcut function' with k=24 as the number of clusters (*i.e.*, the number of principal components explaining 95% of variation). A total of 15 metabolic measures, each representing a separate cluster, was selected for the sensitivity analysis: due to limitations in the availability of the public data (only 87 measures available in all studies), the number of metabolic measures selected for the analysis was smaller than the number of clusters. Red circles indicate the metabolomic measures that showed significantly different effects of visceral fat vs. type 2 diabetes, myocardial infarction, or ischemic stroke ($p_{diff}<0.05$). The shaded area shows the 95% confidence interval for the line indicating the linear fit between the metabolomic profiles.

Supplementary References

1. Ahola-Olli A V., Mustelin L, Kalimeri M, et al. Circulating metabolites and the risk of type 2 diabetes: a prospective study of 11,896 young adults from four Finnish cohorts. *Diabetologia*. 2019;62(12):2298-2309. doi:10.1007/s00125-019-05001-w

2. Holmes M V., Millwood IY, Kartsonaki C, et al. Lipids, Lipoproteins, and Metabolites and Risk of Myocardial Infarction and Stroke. *J Am Coll Cardiol*. 2018;71(6):620-632. doi:10.1016/j.jacc.2017.12.006
