# Supplementary material for: Visceral adiposity is associated with metabolic profiles predictive of type 2 diabetes and myocardial infarction
Source: Commun Med (Lond). 2022 Jul 1;2:81. doi: 10.1038/s43856-022-00140-5 (PMC9249739; doi:10.1038/s43856-022-00140-5)
Supplement: Supplementary file 4 — Reporting Summary [file 43856_2022_140_MOESM4_ESM.pdf]

nature research

Corresponding author(s): Zdenka Pausova

Last updated by author(s): Feb 15, 2022

Reporting Summary

Nature Research wishes to improve the reproducibility of the work that we publish. This form provides structure for consistency and transparency in reporting. For further information on Nature Research policies, see our Editorial Policies and the Editorial Policy Checklist.

Statistics

For all statistical analyses, confirm that the following items are present in the figure legend, table legend, main text, or Methods section.

n/a	Confirmed	
☐	☒	The exact sample size (*n*) for each experimental group/condition, given as a discrete number and unit of measurement
☐	☒	A statement on whether measurements were taken from distinct samples or whether the same sample was measured repeatedly
☐	☒	The statistical test(s) used AND whether they are one- or two-sided<br>*Only common tests should be described solely by name; describe more complex techniques in the Methods section.*
☐	☒	A description of all covariates tested
☐	☒	A description of any assumptions or corrections, such as tests of normality and adjustment for multiple comparisons
☐	☒	A full description of the statistical parameters including central tendency (e.g. means) or other basic estimates (e.g. regression coefficient) AND variation (e.g. standard deviation) or associated estimates of uncertainty (e.g. confidence intervals)
☐	☒	For null hypothesis testing, the test statistic (e.g. *F*, *t*, *r*) with confidence intervals, effect sizes, degrees of freedom and *P* value noted<br>*Give P values as exact values whenever suitable.*
☒	☐	For Bayesian analysis, information on the choice of priors and Markov chain Monte Carlo settings
☐	☒	For hierarchical and complex designs, identification of the appropriate level for tests and full reporting of outcomes
☐	☒	Estimates of effect sizes (e.g. Cohen's *d*, Pearson's *r*), indicating how they were calculated

Our web collection on statistics for biologists contains articles on many of the points above.

Software and code

Policy information about availability of computer code

Data collection	No software was used for data collection
Data analysis	All analyses were conducted using R libraries described in Methods and, thus, this paper does not report original code. The exact R commands used are available upon request.

For manuscripts utilizing custom algorithms or software that are central to the research but not yet described in published literature, software must be made available to editors and reviewers. We strongly encourage code deposition in a community repository (e.g. GitHub). See the Nature Research guidelines for submitting code & software for further information.

Data

Policy information about availability of data

All manuscripts must include a data availability statement. This statement should provide the following information, where applicable:

- Accession codes, unique identifiers, or web links for publicly available datasets
- A list of figures that have associated raw data
- A description of any restrictions on data availability

Individual level data is available upon request from the Saguenay Youth Study. All summary statistics are available within the related supplemental files.

Field-specific reporting

Please select the one below that is the best fit for your research. If you are not sure, read the appropriate sections before making your selection.

☒ Life sciences ☐ Behavioural & social sciences ☐ Ecological, evolutionary & environmental sciences

For a reference copy of the document with all sections, see nature.com/documents/nr-reporting-summary-flat.pdf

Life sciences study design

All studies must disclose on these points even when the disclosure is negative.

Sample size	No sample size calculation was performed. Our sample size (938 adolescents and 507 adults) was predetermined by the size of the Saguenay Youth Study. All individuals with both MRI-quantified visceral fat and NMR-based metabolomics data were included in this study. The sample size was sufficient to demonstrate multiple significant results after correction for multiple testing.
Data exclusions	Since our study investigates the association between circulating lipids and visceral fat, individuals with self-reported lipid disease (15 adolescents and 14 adults) or on lipid-lowering medications (3 adolescents and 77 adults) were excluded from the analyses. Our final sample included 938 adolescents and 507 adult parents.
Replication	We describe in the study limitations that replication of our findings in other populations would be of high value. To our knowledge, however, there are no other cohorts having both NMR-based metabolomics data and MRI-quantified visceral fat volume measured in the general population and, especially, in adolescents.
Randomization	Prior to association testing, all variables were adjusted for age, sex, age-by-sex interaction, genetic relatedness and shared family environment; visceral fat and subcutaneous fat were additionally adjusted for height. The sex-specific subsamples were adjusted for age, height, genetic relatedness, and shared family environment.
Blinding	No groups allocation was done in the present study.

Reporting for specific materials, systems and methods

We require information from authors about some types of materials, experimental systems and methods used in many studies. Here, indicate whether each material, system or method listed is relevant to your study. If you are not sure if a list item applies to your research, read the appropriate section before selecting a response.

Materials & experimental systems

n/a	Involved in the study	
☒	☐	Antibodies
☒	☐	Eukaryotic cell lines
☒	☐	Palaeontology and archaeology
☒	☐	Animals and other organisms
☐	☒	Human research participants
☒	☐	Clinical data
☒	☐	Dual use research of concern

Methods

n/a	Involved in the study	
☒	☐	ChIP-seq
☒	☐	Flow cytometry
☒	☐	MRI-based neuroimaging

Human research participants

Policy information about studies involving human research participants

Population characteristics	The studied sample consisted of 938 adolescents (average age 15 years, 48% male) and 507 adults (average age 49 years, 45% male). The prevalence of overweight or obesity was 68% in adults and 28% in adolescents, which is similar to the Canadian population at large. The average BMI was 27.9 kg/m2 in adults and 21.8 kg/m2 in adolescents.
Recruitment	The participants were recruited via high schools from the Saguenay Lac Saint-Jean region of Quebec, Canada.
Ethics oversight	The study was approved by the Research Ethics Boards of the Chicoutimi Hospital in Chicoutimi, Canada and of the Hospital for Sick Children in Toronto, Canada.

Note that full information on the approval of the study protocol must also be provided in the manuscript.
